# Photodynamic Therapy, Probiotics, Acetic Acid, and Essential Oil in the Treatment of Chronic Wounds Infected with *Pseudomonas aeruginosa*

**DOI:** 10.3390/pharmaceutics15061721

**Published:** 2023-06-13

**Authors:** Jaeson D. Chin, Lei Zhao, Trenton G. Mayberry, Braydon C. Cowan, Mark R. Wakefield, Yujiang Fang

**Affiliations:** 1 Department of Microbiology, Immunology & Pathology, Des Moines University, Des Moines, IA 50312, USA; 2 The Department of Respiratory Medicine, The Second People’s Hospital of Hefei and Hefei Hospital Affiliated to Anhui Medical University, Hefei 230002, China; 3 Department of Surgery, University of Missouri School of Medicine, Columbia, MO 65212, USA; 4 Ellis Fischel Cancer Center, University of Missouri, Columbia, MO 65212, USA

**Keywords:** chronic wounds, photodynamic therapy, probiotics, acetic acid, *Pseudomonas aeruginosa*

## Abstract

As a prevalent medical problem that burdens millions of patients across the world, chronic wounds pose a challenge to the healthcare system. These wounds, often existing as a comorbidity, are vulnerable to infections. Consequently, infections hinder the healing process and complicate clinical management and treatment. While antibiotic drugs remain a popular treatment for infected chronic wounds, the recent rise of antibiotic-resistant strains has hastened the need for alternative treatments. Future impacts of chronic wounds are likely to increase with aging populations and growing obesity rates. With the need for more effective novel treatments, promising research into various wound therapies has seen an increased demand. This review summarizes photodynamic therapy, probiotics, acetic acid, and essential oil studies as developing antibiotic-free treatments for chronic wounds infected with *Pseudomonas aeruginosa*. Clinicians may find this review informative by gaining a better understanding of the state of current research into various antibiotic-free treatments. Furthermore. this review provides clinical significance, as clinicians may seek to implement photodynamic therapy, probiotics, acetic acid, or essential oils into their own practice.

## 1. Introduction

### 1.1. Bacterial Infections of Wounds

Long-lasting persistent open wounds are prone to infections by various pathogens, especially bacteria. Specifically, bacterial infections are common and pose a challenge to those suffering from chronic wounds. In some of these infections, the bacteria will form a biofilm that acts as a survival mechanism against the human immune system and antibiotic treatments [1]. When bacteria form a biofilm, the infection will have the potential to become chronic and untreatable. Therefore, a strong relationship exists between bacteria with a biofilm growth phenotype and clinically chronically infected wounds [2,3]. Biofilm is a physical matrix that connects clusters of bacteria to each other and/or to a surface. Protected by the biofilm matrix, bacteria can hide from the host’s immune system and cause tissue damage, reduce rates of metabolism and cell division, and eventually become resistant to antimicrobial treatments [3]. Many biofilm-forming bacteria commonly infect wounds [4]. *Pseudomonas aeruginosa* (*PA*) is a Gram-negative biofilm-forming bacteria with antibiotic resistance that is commonly found in chronic wounds [5]. A 13-year prospective cohort study showed increased mortality for patients with a *PA* infection compared to infections by other biofilm-forming bacteria such as *Staphylococcus aureus* (*S. aureus*) [6]. It has also been determined that *PA* accounts for 10.8% of skin and soft tissue infections acquired in a North American hospital [7]. Treatment for *PA* infections will be the focus of this review. 

### 1.2. Chronic Wounds

Chronic wounds, otherwise known as hard-to-repair wounds, appear in approximately 2% of the world’s hospitalized patient population. In the United States alone, over 6 million people are affected by chronic wounds or chronic ulcers, which has a substantial impact on mortality and morbidity [8]. Chronic ulcers are commonly caused by diabetes, vascular disease, or neuropathy, and frequently affect the elderly population [9]. These wounds encumber the healthcare system and require constant treatment and management. If a wound is not healed within 6 to 8 weeks, it is classified as chronic [10,11,12]. This paper will explore current and developing antibiotic-free *PA* treatments in chronically infected wounds. Clinical diagnosis of *PA*-infected wounds can be made through visual and sometimes olfactory cues [13]. Cultural-dependent analyses are often necessary to confirm *PA* infection. Recent research has shown fluorescence imaging as a developing technique that proves rapid detection of *PA* in chronic wounds. This may be valuable in cases in which other identifying clinical symptoms do not appear [14]. 

### 1.3. Antibiotic Resistance

**Figure 1:**
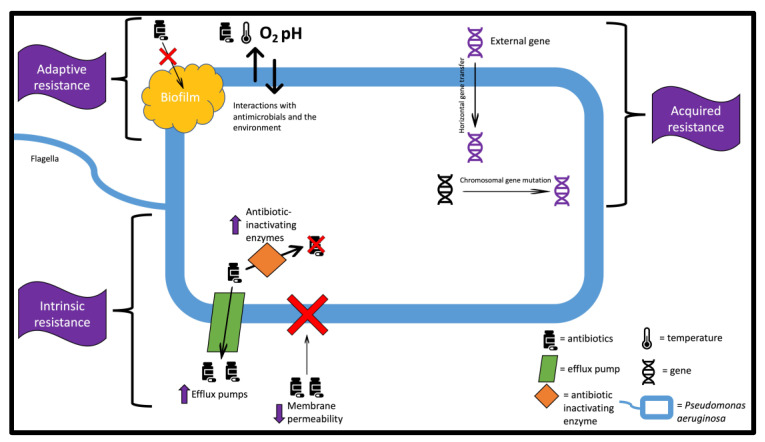
Types of *PA* antibiotic resistance, including intrinsic, adaptive, and acquired. Increased expression of efflux pumps is one mechanism of intrinsic resistance. These pumps work to get rid of harmful substances such as antibiotics from the cell’s internal environment. *PA* also synthesizes enzymes that perform chemical alterations on the antibiotic, leading to its inactivation. Other enzymes hydrolyze antibiotics leading to their destruction. The cell may also induce alteration of porin membrane proteins leading to decreased membrane permeability. This decreased permeability prevents antibiotics from entering the cell. Adaptive resistance occurs through rapid alterations of the cell due to environmental changes and the presence of antibiotics. Biofilm formation, which prevents diffusion of antibiotics into the cell, can also trigger adaptive resistance. Acquired resistance is when the bacterium develops resistance through genetic modification either through horizontal gene transfer or chromosomal gene mutations.

Presently, to prevent and/or treat bacterial infections, medical professionals usually use antibiotics as their primary weapons. The first antibiotic was developed in 1910. Since then, antibiotics have greatly impacted medicine and have led to lifesaving discoveries, such as that of penicillin in 1928. However, due to the ability of bacteria to evolve, the current-day medical world has been facing an antibiotic resistance crisis due, at least in part, to the widespread misuse and over-prescription of antibiotics. The situation has become so severe that some infections are untreatable through antibiotics. The UK government has predicted that 10 million people a year will perish due to drug-resistant infections by the year 2050 [15]. Within the context of chronic wounds, antimicrobial resistance increases the burden of wound care and is strengthened by the microenvironment, which promotes biofilm formation. In a study performed in 2016, antibiotic-resistant strains were present in half of 110 patients’ chronic wounds [16]. *PA* commonly displays antimicrobial resistance, complicating the treatment for this pathogen. Alongside skin infections, *PA* also is known to cause pneumonia, gastrointestinal infections, dermatitis, urinary tract infections, cystic-fibrosis-related respiratory infections, burn wound infections, bone and joint infections, and infections in immunocompromised patients [17,18]. Due to *PA*’s ability to adapt to threats, commonly used antibiotic treatments such as cephalosporins and carbapenems have been rendered ineffective in some strains of this pathogen, leading to the creation of multidrug-resistant strains of *PA* [19]. The overall mortality rate for patients infected with the *PA* pathogen was found to be 31%, while those with multidrug-resistant strains suffered a mortality rate of 67% [17]. *PA* has three main mechanisms for obtaining antimicrobial resistance: intrinsic resistance, acquired resistance, or adaptive resistance [20]. Intrinsic resistance takes place when a microorganism develops the ability to resist antimicrobial agents by decreasing outer membrane permeability, expressing MDR efflux pumps, and producing antibiotic-inactivating enzymes [21]. Acquired resistance forms when a bacterium acquires external genes that lead to resistance through horizontal gene transfer and chromosomal gene mutations [22]. Adaptive resistance occurs when bacteria are exposed to antimicrobial agents or environmental stressors leading to adaptive changes by the pathogen [23]. Biofilm, commonly formed by *PA,* provides further resistant properties [3]. Figure 1 displays the three types of resistance occurring in *PA.* Overall, *PA* exists as an opportunistic pathogen that may require alternative treatments to fight off.

In recent years, photodynamic therapy, probiotics, acetic acid, and essential oils have been researched as potential and innovative antibiotic-free treatments for chronic wounds, and for *PA*-infected chronic wounds specifically. These four methods of treatment were chosen for this review manuscript as each presents a unique way to treat infected wounds. While these four fields of research are not a comprehensive collection of new innovations, the authors of this manuscript believe that they demonstrate promising results and present the diversity of current research interests in the search for antibiotic-free treatments.

## 2. Photodynamic Therapy

Photodynamic therapy (PDT) uses light energy at a specific wavelength to activate photosensitizers within their target. In the case of infections, PDT aims to create reactive oxygen species that will kill the bacterial pathogen and disrupt its biofilm. Although the concept of PDT has existed since the 1900s, only recently has antimicrobial photodynamic inactivation seen a newfound sense of interest with the rise of multidrug-resistant strains of pathogens [24,25]. Since PDT uses irradiation as its treatment method, it has been investigated as a useful antibiotic-free technique that does not lead to novel resistant bacteria [26,27,28]. Besides treating chronic wound infections, PDT has been investigated in the field of dental care, and to treat diseases such as cystic fibrosis, chronic sinusitis, cancer, and more [29,30].

**Figure 2:**
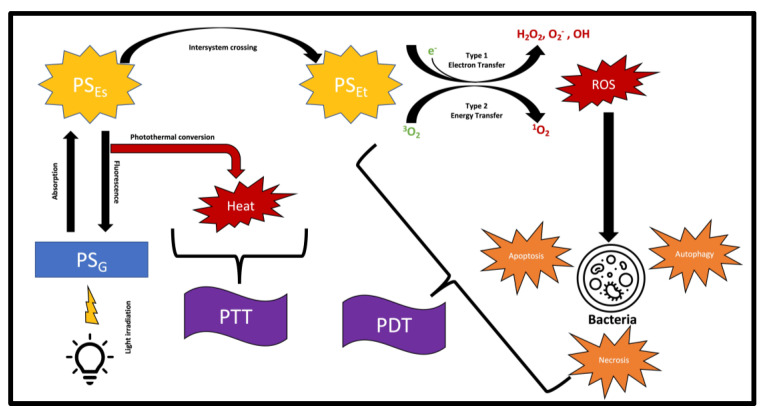
A schematic displaying the mechanism behind photodynamic therapy (PDT), and photothermal therapy (PTT). A light source at a specific wavelength irradiates a photosensitizer (PS) from its ground state (PS_G_) to an excited singlet state (PS_Es_). The PS_Es_ is then transformed into an excited triplet state (PS_Et_) through intersystem crossing. Returning to PS_G_ results in fluorescence and photothermal conversion. Heat produced from this reaction is used in PTT. From PS_Et,_ a reactive oxygen species (ROS) is created through either type 1 or type 2 chemical reactions. A type 1 mechanism, known as electron transfer, includes the creation of free radicals and radical ions through electron transfer reactions. A type 2 mechanism, called energy transfer, involves a molecule of oxygen which is used to generate a singlet oxygen. ROS created from either reaction then act on targets such as bacteria, viruses, protozoa, and cancerous cells, causing damage and death. Bacteria cells are destroyed through apoptosis, autophagy, or necrosis pathways.

The mechanism behind PDT involves the excitation of electrons within the photosensitizer molecule which then collides with an oxygen molecule (O_2_) forming a singlet oxygen [31]. It is also possible for the photosensitizer electron to perform electron transfer reactions with oxygen, forming reaction oxygen species [24]. These excited oxygen molecules then act by inflicting photodynamic damage on nearby cells [32]. Affected cells will die via three main pathways; apoptosis, necrosis, or autophagy-associated cell death. The subcellular location and the concentration of the photosensitizer is thought to influence which one of the three cell death mechanisms the cell will undergo [24]. PDT’s mechanism of action is shown in Figure 2.

To treat *PA* infections in chronic skin ulcers, PDT research has mostly been limited to small studies or case studies. In vivo studies prove to be difficult due to the instability of *PA* animal models [33]. However, multiple studies have explored the safety and effectiveness of PDT with a 5-aminolevulinic acid (ALA) photosensitizer. ALA, known as a “prodrug”, is a biosynthetic precursor to protoporphyrin IX which ultimately functions as a photosensitizer. ALA is converted to protoporphyrin IX metabolically once it enters the body [34]. ALA can be applied topically, making it an attractive option for dermatological interventions such as treating chronic wounds. A study highlighted that the effectiveness of ALA/PDT treatment relies on the ability of ALA to enter the cell and then be converted to protoporphyrin IX. This process is different across bacterial species, meaning that bacterial-specific research may be crucial for future investigation into this subject [35].

A higher concentration of ALA (20%) has been shown to produce antimicrobial effects, reducing bacterial levels in infected wounds [36,37]. The use of a 20% ALA photosensitizer also reduced the area of wounds and promoted wound healing [36,37]. However, higher concentrations of ALA have been shown to produce negative side effects associated with the treatment, including acute pain [36,37,38,39]. With a lower concentration of ALA, pain and other adverse side effects seem to be eliminated [40,41]. The effectiveness of using a low concentration of ALA in PDT still has much room for investigation. One study used 2% ALA and a PDT light with a wavelength of 560 nm–780 nm to treat three patients with chronic lower leg ulcers. The 2% ALA/PDT treatment eliminated bacteria from the wound, leading to wound healing after one to three treatments. The patients also noted minimal side effects [40]. This study was based on an exceedingly small cohort of patients. Future studies could use a larger sample size to further validate the use of a low concentration of ALA to eliminate *PA* and heal chronic wounds. In another study, ALA (0.5%) and an EDTA-2Na ointment were applied to human skin ulcers [41]. EDTA-2NA works to increase the production of protoporphyrin IX, making the PDT more effective [42]. This combination was activated by a 410-nm LED light source on consecutive days until the infection site disappeared and the wound healed. Seven patients with skin ulcers infected with *S. aureus* and *PA* received the treatment. Results showed a reduction in the ulcer areas of all patients. Decreased white blood cell counts in four patients after 4 weeks of treatment were also observed. This result suggested that their infections were eliminated. However, only three of the seven patients showed a decreased bacterial colony count. Overall, PDT with 0.5% ALA effectively decreased the open wound area. The researchers concluded that the treatment was effective against infection and did not cause any serious side effects [41]. Results from these studies have been summarized in Table 1. More research into the most efficient concentration of ALA and wavelength of light is needed to optimize and balance potential side effects with the efficacy of the treatment’s ability to treat bacterial infection and promote wound healing. A high concentration of ALA, such as 20%, seems to be the most effective in eliminating bacterial infections. However, the pain levels associated with such concentrations prevent it from being an optimal treatment method and leaves room for improvement. On the other hand, a lower concentration of ALA produces no adverse side effects, but the effectiveness of the treatment is questionable. Low concentrations of ALA only seem to be effective with help from other compounds such as EDTA-2Na, but even then, results are inconsistent. Research into compounds that can increase the effectiveness of ALA is an intriguing area of study. Such compounds may act to increase ALA’s uptake into cells or increase the metabolic conversion of ALA to protoporphyrin IX. The CAALA, or Complex Augmentation of ALA, regimen has been developed as a way to increase protoporphyrin content in glioblastoma cells. CAALA consists of ciprofloxacin, iron chelator deferiprone, antimetabolite 5-FU, and the xanthine oxidase inhibitor febuxostat [43]. Such drugs may also increase ALA’s effectiveness in a chronic wound context and could be a subject of future endeavors.

Besides ALA, other compounds have been investigated for their use as a photosensitizer in eliminating the *PA* pathogen. ALA is compared to methylene blue and indocyanine green in Table 2.

Ideally, photosensitizers should be a storage-stable single pure compound with low manufacturing costs. It would be expected for the compound to be without toxicity while displaying rapid clearance from normal tissue [24,45]. Furthermore, a photosensitizer used to treat bacterial infections should selectively target pathogens and avoid uptake into healthy cells. Reactive oxygen species created by PDT are also toxic to normal healthy cells. As such, PDT must be optimized in a way so that it can be safely administered around nearby healthy tissue.

Synthetic dyes such as methylene blue and indocyanine green carry a cationic charge. These positively charged photosensitizers have a high affinity and bind easily to Gram-negative and Gram-positive bacteria, thus providing selectivity [46]. Porphyrins such as ALA usually accumulate in high quantities in bacteria [46], but it is unclear whether this holds true for *PA*. Overall, using selective photosensitizers seems to be the most effective way to limit PDT’s effects on normal cells at this time. Current research into improving the selectivity of photosensitizers includes exploring the functionalization of these molecules. For example, one study discovered that the addition of a tertiary ammonium to methylene blue increased its attachment and uptake by Gram-negative and Gram-positive bacterial cells [47].

Methylene blue belongs to a group of phenothiazinium dyes and is used in different areas of clinical medicine [48]. One study demonstrated the use of methylene blue to reduce *PA*-formed antibiotic-resistant biofilms. The study used an endotracheal tube in vitro model and found a >99.9% reduction in biofilm size after a single treatment [49]. Although this study did not use methylene blue to treat infected chronic wounds, it still shows the effectiveness of methylene blue against *PA.* Another study used methylene blue PDT to eliminate clinical isolates of extensively drug-resistant *PA* [50]. As multidrug-resistant strains of bacteria are common in chronic wounds [51], therapies that can treat such strains are especially important. Both experiments utilized in vitro models. This is a limitation when trying to connect these studies’ results to the clinical application of chronic wounds. For example, the application of methylene blue to the target would be different in vivo. Furthermore, the environment of a chronic wound within a human body affects its overall properties and its healing process [52,53]. The use of methylene blue PDT has been explored in various small-scale case studies. These studies have found methylene blue to be an effective photosensitizer to treat infected chronic wounds without adverse side effects [54,55,56,57]. Larger-scale studies with proper controls would help strengthen these conclusions. Furthermore, some of the patients in one of the studies were administered antibiotic treatment alongside PDT [57]. However, evidence from numerous studies supports the effective elimination of bacteria without antibiotic therapy [54,55,57]. Further advantages and disadvantages pertaining to methylene blue are summarized in Table 2. For methylene-blue-mediated PDT to be a standalone treatment for *PA* infections of chronic wounds, further studies are needed to clarify its utility in the absence of antibiotics. Moreover, one study found that toluidine blue, a compound similar to methylene blue [58], exhibited greater bacterial activity [59]. Other photosensitizers similar to methylene blue may be worth investigating.

Indocyanine green is another photosensitizer that is a part of the dye family. It has been used in a variety of medical fields since the 1950s due to its broad application and low risk to patients. Recently, new functions of the dye have emerged including its use in wound-related care and PDT [60]. Several in vitro studies have documented the effectiveness of indocyanine-green-mediated PDT to successfully kill *PA* cells [61,62,63]. Not surprisingly, the bactericidal effect on *PA* cells has been found to be dependent on the concentration of indocyanine green used [63]. However, in contrast to ALA, there seem to be no adverse side effects of higher doses of indocyanine green [63,64]. Other studies have found indocyanine green to be effective in killing *S. aureus,* a biofilm-forming bacterium similar to *PA* [65,66]. Although there is a lack of in vivo research, the results of these in vitro studies show its clinical potential. Moreover, indocyanine green has a high absorption wavelength compared to other photosensitizers such as ALA and methylene blue. As higher wavelengths of light penetrate deeper, indocyanine-green-mediated PDT can reach tissues that otherwise may be inaccessible with a photosensitizer with a lower absorption wavelength [24,61,63]. The mechanism behind indocyanine-green-mediated PDT and its wound healing is still unknown. A study has shown this process is related to cystic fibrosis transmembrane conductance regulators (CFTRs) and their involvement in cell migration [67]. Overall, as indocyanine-green-mediated PDT shows promise as an effective treatment for *PA* wounds, more in vivo studies are needed to explore its application in clinical medicine.

Natural photosensitizers such as curcumin and *Curcuma xanthorrhiza* extract have the ability to induce a photodynamic reaction with a 405 nm light [68]. Although these molecules have not yet been used in PDT studies related to chronic wounds, they have been shown to exhibit antibacterial properties [69,70,71]. Additionally, they are activated at a short wavelength. While longer wavelengths of light are generally able to penetrate tissue at greater depths, a shorter wavelength of light carries higher energy and may be able to treat superficial wounds more effectively [68].

Chlorophyll molecule derivatives are another category of natural photosensitizers that can potentially play a role in the treatment of chronic wounds. Natural chlorophyll molecules, specifically chlorophyll *a* and bacteriochlorophyll *a*, are photosensitizer derivatives that absorb light at a wavelength of 700–800 nm [72]. Chlorophyll molecules display high antimicrobial properties and can efficiently eliminate bacteria through irradiation [73,74]. A study also discovered a lack of cytotoxic effects in healthy cells when using a chlorophyll-rich *Tetragonia tetragonoides* extract [73]. Photosensitizers such as chlorophyll *a* are relatively cost-effective, easily eliminated from the body, and can absorb penetrating light (wavelengths of 700–800 nm) [72,75,76]. As such, chlorophyll-derived photosensitizers may be efficient when treating deep or large wounds [72,76]. There is a lack of literature exploring the use of chlorophyll to treat *PA* infections. Despite this, chlorophyll-based PDT has been shown to be effective in other settings, such as eliminating oral, acne-, and food-related bacteria [77,78,79,80]. As such, chlorophyll/PDT may be a potential avenue of research for therapies targeting chronic wounds.

A few studies demonstrated the use of chlorin-based PDT to produce viable antimicrobial activity and inhibition of bacterial growth in animal models with wound infections [74,81,82]. It is believed that PDT treats chronic wounds by eliminating harmful bacteria on the surface of the ulcer through disruption of bacterial biofilm. Chlorin photosensitizers allow for better penetration into the bacteria and sufficient ROS generation to eliminate *PA* [74]. Furthermore, chlorin-mediated PDT also decreases hyperinflammatory cytokine responses in *PA*-infected wounds and decreases bacteria protease activity leading to the downregulation of IL-6 and TNF-α (critical inflammatory mediators in chronic wounds) [81]. Therefore, chlorin-based PDT may be beneficial in treating chronic wounds through multiple mechanisms (directly eliminating bacteria and reducing the hyperinflammatory response of *PA*).

Although the mechanisms behind the action of PDT on wounds are still being explored, other studies have also reported various connections with the inflammatory microenvironment. One study found that PDT was increased in T regs, plasmacytoid dendritic cells, and MHCII-positive dermal DCs. The study also showed that increased TGF-beta is correlated with a reduction in wound size [39]. Another study corroborated these findings demonstrating an increase and then decrease in inflammatory factors TNF-α and IL-1β. A gradual increase in growth factors TGF-β-1 and VEGF was also reported. Ultimately, ALA-PDT treatment of *PA* in skin wounds works, at least in part, by affecting the wound’s granular tissue formation, angiogenesis, and collagen regeneration and remodeling. It also changes the activation state of macrophages [44].

**Table 2:** Three common photosensitizers used in PDT for chronic wounds and their associated advantages and disadvantages.

Photosensitizers Used in PDT for Chronic Wounds	Advantages	Disadvantages	Sources
Methylene blue dye (absorption wavelength of 630–690 nm [48])	Safe, cheap, approved for human intravenous use, readily available [54,55,56]; Elimination of chronic wound infection [54,55,57]; Reduction and healing of wounds [54,55,56,57]; No side effects when treating chronic wounds [54,55,56]; Kills multidrug-resistant bacteria including PA in vitro [49,50]	Serious adverse reactions with the central nervous system when administered to patients who take psychiatric medications that affect the serotonin system [83,84,85]	[48,49,50,54,55,56,83,84,85]
Indocyanine green dye (absorption wavelength of 600–900 nm [86])	High absorption wavelength allows for bactericidal and photobiological effects of treatment to reach deeper tissues/wounds [24,61,63]; Indocyanine-green-mediated PDT has bactericidal activity against multidrug-resistant strains of PA in vitro [61,62,63]; Can be used in PDT/PTT combination therapy to treat bacterial infections [62,87,88]; Less expensive than ALA [89]; Low toxicity and little to no known side effects [63,64]	Lack of in vivo studies investigating indocyanine green’s use in PA-infected chronic wounds	[24,61,62,62,63,64,86,87,88,89]
5-aminolevulinic acid (ALA) tetrapyrrole structure (absorption wavelength of 400–630 nm [90])	Can be applied topically and is easily absorbable by the skin [91]; ALA-mediated PDT reduces bacterial infection in chronic wounds [36,37,40]; Reduction and healing of wounds [36,37,39,40,41]; No reoccurrence of infection [37,40]	Adverse side effects were reported especially with higher concentrations of ALA [36,37,39,41,92,93]; Topical application does not penetrate into tissue, making deep wounds hard to treat [93,94]	[36,37,39,40,41,90,91,92,93,94]

PDT has also been used in combination with other therapies to treat bacterial infections. Photothermal therapy (PTT), a similar light-based therapy, has been used alongside PDT. Similar to PDT, PTT contributes to disrupting the biofilm caused by bacterial infections. However, it does this through thermal destruction in contrast to the ROS that PDT generates [95,96]. PTT’s mechanism of action is illustrated in Figure 2. A study found success by using superparamagnetic iron oxide nanoparticles (SPIONs) and the photosensitizer indocyanine green to destroy the biofilm of *PA*. By performing a 10 min irradiation with a laser at 808nm, PDT combined with PTT resulted in the complete elimination of *PA* cells [87]. Indocyanine-green-based PTT/PDT has also been shown to work against bacteria similar to *PA.* A study used this dual therapy with mesoporous polydopamine nanoparticles to destroy an *S. aureus* biofilm [88]. Another study utilized PTT, PDT, and molybdenum trioxide nanodots to treat wounds infected with *E. coli* and *S. aureus.* The study used one light source of 808 nm wavelength to activate both PDT and PTT [97]. Other nanoparticles, such as nanographene oxide, copper sulfide, and iron oxide, can also act as photosensitizers during PTT or PDT [98,99,100]. Despite the use of nanomaterial in PTT/PDT research, it should be noted that nanomaterials can accumulate in healthy tissue. Build-up of these materials is hard to identify and eliminate, and can lead to cytotoxicity [101]. Further research is needed to probe into the effects of nanomaterials and their potential hazards when used for PTT/PDT.

Previous research has examined PDT alongside nanomaterials such as inorganic salts. The addition of salts such as potassium iodide, sodium thiocyanate, sodium azide, and sodium nitrite improved the effectiveness of PDT to kill off bacterial infections in animal models [102]. Silver nanoparticles have the ability to act as photosensitizers or reactive oxygen species under irradiation [103]. Additionally, they have been found to inhibit the biofilm formation of *PA* [104,105]. Interestingly, silver nanoparticles have been combined with photosensitizer methylene blue to increase the antimicrobial effect of PDT [106]. As such, silver nanoparticles may provide synergistic interactions that could improve PDT and should be investigated in this field further.

Another combinational therapy includes the use of PDT with a pulsed electric field. It has been demonstrated to be a potent duo when attacking bacterial biofilms [107]. An electric field functions by causing more extensive chemical reactions to occur, thus leading to the creation of more reactive oxygen species. Additionally, it is hypothesized that merging PDT with a pulsed electric field (PEF) increases photosensitizer permeabilization. Therefore, a PEF and PDT concurrent treatment leads to the elimination of biofilm bacterial infections through two effective mechanisms [107]. Moreover, a wireless electroceutical dressing that emits an electrical field was found to disrupt the biofilm of *PA* infections in chronic wounds [108]. Other studies have used pulsed electric fields as a method to make biofilm bacteria more susceptible to antimicrobial agents [109,110]. Pulsed electric fields can also be used to reduce bacterial attachment to a steel surface [111].

Recent research has shown the ability of several photosensitizers as well as the effectiveness of PDT to fight *PA* bacterial infections, as well as multidrug-resistant strains of *PA*. With interest growing in finding alternatives to treat infected chronic wounds, PDT has a significant amount of evidence supporting its efficacy in eliminating harmful bacteria such as *PA* and promoting the healing of long-lasting wounds. Additionally, PDT will not lead to any new drug-resistant properties in bacteria [26,27,28]. Several photosensitizers show promise as being safe and effective compounds to use in PDT. Future research in this field may include experimenting with various photosensitizers and concentrations to further assess the side effects and effectiveness of photodynamic therapy while also continuing to explore the mechanisms behind PDT. Moreover, the application of various therapies or other compounds alongside PDT is an area of investigation that may lead to innovative ways to improve efficacy.

## 3. Probiotics

Probiotics are microorganisms naturally found in the human body. The World Health Organization (WHO) defines probiotics as “live microorganisms which, when administered in adequate amounts, confer a health benefit to the host” [112]. Probiotics, in general, are a newer field, as the WHO defined the word recently in the year 2000 [112]. As a growing topic, probiotics have seen an increase in popularity due to their potential benefits in balancing the gut microbiota and for its respiratory, gastrointestinal, oral, vaginal, and possibly chronic wound applications [113,114,115,116].

Although the topical antibacterial actions of probiotics are not fully understood, they likely act under various mechanisms [117]. Through the production of exopolysaccharides, probiotic bacteria enhance immunity by stimulating immune cells such as macrophages and lymphocytes [118,119]. Another mechanism used by probiotics includes the regulation of antimicrobial peptides which play a role in the skin’s microflora, cell proliferation, and angiogenesis [120,121,122,123]. Probiotics have also been found to decrease pathogenic bacteria through specific mechanisms depending on the bacterial species [124,125,126].

Encapsulation of probiotics has seen recent innovation and interest. Probiotics are usually incompatible with antibiotics as they decrease their therapeutic ability. When being administered alongside antibiotics, encapsulation can serve to protect the probiotics [127]. Encapsulation can also protect probiotics from the immune system and control them to a localized area [128,129]. Even further, encapsulation has exhibited antibacterial activity in vitro and accelerated wound healing in an in vivo mouse model [128]. Several encapsulation biomaterials are the subject of investigation. A hydrogel encapsulation provides a beneficial environment for tissue repair and regeneration while also having the ability to absorb excessive wound secretions [128,130]. Another encapsulation method is the use of a biofilm-based encapsulation which can be used to administer probiotics. This biofilm-based encapsulation is build around alginate, an extracellular polymeric component of pseudomonas biofilm. Alginate is an anionic polysaccharide that can be cross-linked with cations to form hydrogels. The encapsulate works to protect the probiotics while still allowing their outward diffusion [127]. Alginate encapsulation has been approved by the U.S. Food and Drug Administration for its use as a food additive. Besides its potential application in wound healing, it has also been researched as a method to protect probiotics in food products and in the gastrointestinal tract [131].

*Lactobacillus plantarum* (*L. plantarum*) is one type of probiotic that has been studied frequently in the treatment of bacterially infected wounds. Several animal model studies have proved *L. plantarum* as an effective treatment for *PA* [132,133,134,135,136]. The mechanisms behind *L. plantarum’s* antimicrobial effects are still being uncovered. Interestingly, one study demonstrated a possible mechanism involved with signaling molecules called acyl-homoserine-lactones (AHLs). *PA* releases these AHLs, which then partly control the production of elastase and biofilm [132]. Using a burn wound mouse model and in vitro studies, *L. plantarum* has been shown to block the growth of *PA* and inhibit AHLs, thus hindering elastin and biofilm formation [132]. Another study found that *L. plantarum* treatment successfully prevented a septicemic accumulation of *PA* in mouse organs. [135]. It also restricted the induction of TNF-α, IL-6, and IL-10 (important cytokines in the inflammatory response system [137]) suggesting a possible reduction of wound inflammation [135]. A similar study revealed that *L. plantarum* significantly diminished inflammation of the wounds examined in burn rat subjects [133]. In a rabbit model, *L. plantarum* treated and healed *PA*-infected burn wounds. While eliminating the *PA* infection, *L. plantarum* also reduced Type I collagen mRNA concentrations and the total collagen protein accumulation, which can be beneficial in reducing scarring [136]. Other research used a combination of four probiotics including *L. plantarum, Lactobacillus acidophilus, Saccharomyces boulardii,* and *Bifidobacterium lactis* to prevent infection with multidrug-resistant *PA.* The pre-treatment use of *L. plantarum* significantly prolonged the survival of the mice subjects with the multidrug-resistant strain of *PA*. Pre-treatment with *Bifidobacterium lactis* also resulted in significant survival benefits [134]. In line with these results, a controlled study using a burn–sepsis mouse model infected with *PA* and then treated with *L. plantarum* reported a 10% mortality rate for the treatment group and a >90% rate for the non-treated group [135].

Several molecules within *L. plantarum* have been identified as being potentially responsible for the pro-healing and antibacterial properties observed in chronic wounds. These include various antimicrobials, surfactants, anesthetics, and more [138]. It has been proposed that *L. plantarum* interferes with *PA* through suppression of the bacteria’s quorum sensing signaling, adhesion, and biofilm formation. It also disturbs other virulence important factors [138,139]. These studies, however, mainly identified molecules found in antibiotics and then drew conclusions about *L. plantarum*’s actions based on the presence of these molecules. A more in-depth and causal relationship between the identified molecules and their mechanisms of action would help solidify the proposed explanation of *L. plantarum*’s workings. Like many probiotics, the effectiveness of *L. plantarum* against *PA* is likely multifaceted and works through several processes [117,124,125,126,132,135,136,138,139].

Few studies have used *L. plantarum* to treat human-infected chronic wounds. As the mechanism of probiotics and a comprehensive understanding of their side effects are largely unknown, the use of probiotics on human wounds remains largely unexplored. Furthermore, as probiotics are live microorganisms, studies are hard to control and several difficulties may arise when attempting to perform clinical trials [140]. Specifically, environmental factors, host-related factors, and specific conditions may impact the viability, potency, and/or safety of probiotics during experiments [140]. Besides the treatment of chronic wounds, probiotics have been studied in humans or through human-based culture to treat gastrointestinal *PA* infections and to decrease *PA* colonization in gut microbiota [141,142].

Despite the lack of research, a few studies have focused on treating *PA*-infected human chronic wounds with *L. plantarum.* One such study used the topical application of an *L. plantarum* culture to treat chronic infected leg ulcers of both diabetic and non-diabetic patients. These patients saw a decrease in their wounds’ bacterial load while simultaneously seeing a boost in debridement and granulation. The treatment also decreased IL-8 production [143]. However, this study did not report the number of probiotics administered correctly as recommended by the World Health Organization (WHO). The WHO recommends that probiotic doses are reported in colony-forming units (CFU) [144]. While research into the usefulness of *L. plantarum* is important, it is also critical to understand how such a treatment stacks up against similar therapies. As such, a human in vivo study compared *L. plantarum* to silver sulphadiazine (the gold standard for burn wound infections [145,146]) on *PA*-infected second-degree and third-degree wounds. They found that *L. plantarum* provided either the same efficiency or a greater efficiency than silver sulphadiazine [147]. Comparison studies may be a relevant topic of interest, especially when considering *L. plantarum* as a possible antibiotic substitute. Further uses of *L. plantarum* may come from mixing the probiotic with others. A case study detailed the successful treatment of a chronic foot ulcer infected with *K. pneumoniae, P. mirabilis*, and *E. faecalis* using a mixture of *Lactobacillus plantarum, Lactobacillus acidophilus*, and *Streptococcus thermophilus*. This formula was topically applied to the wound three times a week. After one week of treatment, the probiotic mixture stabilized the wound. The wound then began to heal and showed microbiological progress after two weeks [148]. This case study reported using a 100 billion CFU mixture of the three probiotics, but did not specify the composition of the mixture. Furthermore, as this was a singular case report based on one patient, the results of the study should be treated with caution. To the authors’ knowledge, these are the first studies to use *L. plantarum* to treat human-infected wounds. More human-based studies are needed to confirm *L. plantarum*’s utility among current antibiotic-free treatments, especially in the case of *PA* infections.

Other species of the *Lactobacillus* genus may provide similar benefits to the wound-healing process. SYNBIO^®^, a 1:1 mixture of *Lactobacillus rhamnosus* and *Lactobacillus paracasei*, was used to treat isolated bacterial pathogens from chronic lesions. The study also used this mixture on an in vitro model. Treatment in both the isolated bacterial pathogens and in vitro models displayed inhibitory actions by preventing biofilm formation [149]. Other research has been carried out using *Lactobacilli fermentum* strains to fight drug-resistant strains of *PA*. The probiotic provided broad inhibition and anti-biofilm effects against the bacterium [150]. While species of the *Lactobacillus* may be used to eliminate pathogens, they also may work to promote overall wound healing outside of infection control. A study used *Lactobacillus rhamnosus* lysate to accelerate the re-epithelialization of keratinocytes in cutaneous wound healing [151]. Topical application of *Lactobacillus reuteri* engineered with a plasmid encoding chemokine CXCL12 was demonstrated to accelerate wound healing and closure in a mouse model and human skin biopsy specimen [152]. If a wound is not yet infected, treatments such as these may function as preventative therapies that help hasten the wound closure process and prevent infections from happening in the first place. All in all, *L. plantarum* and other *Lactobacillus* species are a potential antibiotic-free treatment for infected wounds that can kill bacterial pathogens or can act to avert future potential infections. A more comprehensive understanding behind the mechanisms of action, including the antimicrobial and wound-healing properties of these various strains of probiotics, would help determine the practical use of each. Furthermore, differences and similarities between the therapeutic effects of different strains would benefit from characterizing each strain’s potential role in treating infected chronic wounds. Given the promising results of these in vitro studies, additional investigations into the clinical use of these probiotics may be warranted.

Probiotics, when combined with innovative wound dressings, can potentially result in advantageous synergies. For example, bacterial cellulose, a biopolymer with a high surface area and absorption-like properties, is made possible by aerobic bacteria [153]. This biomaterial has been tested as a wound dressing, given that it provides an ideal scaffold for probiotics to adhere to and grow onto. Additionally, a one-pot or single-step synthesis method proved to integrate more probiotics into the bacterial cellulose scaffold. The higher number of healthy bacteria then promotes high metabolic and cellular activity in wounds. Called probiotic cellulose, this type of wound dressing has been successful in exhibiting antibacterial properties against *PA* and *S. aureus*, two common pathogens found in chronic wounds [154]. Probiotic cellulose may be a valuable tool to be further developed in the field of antibiotic-free treatments. Because of the success of the one-pot method, this treatment is fast and inexpensive to produce, meaning it could be easily scaled for mass consumption [154].

Most of the probiotic studies mentioned in this manuscript do not include analysis of the long-term viability and survival of probiotic treatments. Instead, many of the studies replenished the colony of probiotics by introducing new batches to the treatment site. Future explorations into this field may include finding methods to improve the environment for probiotics in infected wounds. Probiotics may thrive in ideal conditions which can be influenced by temperature, pH, availability of nutrients, and growth factors.

**Figure 3:**
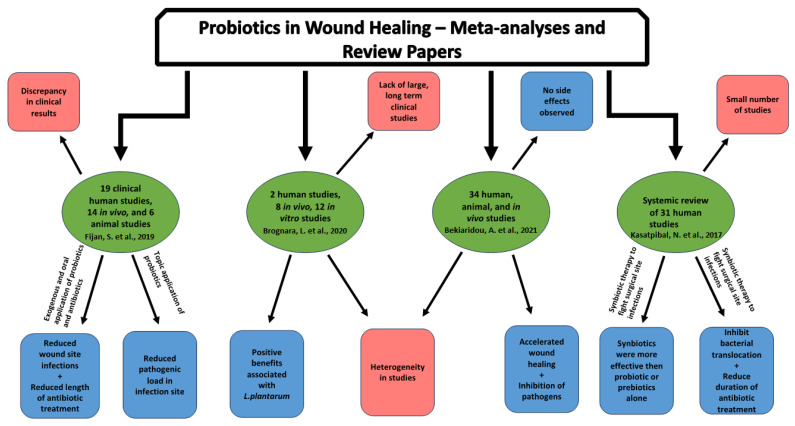
Schematic showing relevant meta-analyses and review papers dealing with probiotics in wound healing. Shown in blue are positive conclusions drawn from the studies while pink displays negative conclusions [4,155,156,157].

Probiotics and their antimicrobial role in wound healing has been researched through numerous animal studies, human studies, meta-analyses, and review papers. As such, systemic literary reviews have recently been performed regarding these studies [4]. One systemic literary review found a discrepancy in the clinical results of probiotics, leading to the questioning of probiotics’ mechanism during wound healing. Despite this, they concluded that probiotics show great potential for reducing wound infections and pathogen loads, whether through exogenous, oral, or topical applications. Further research, specifically on the use of topical antibiotic treatments, was noted to be needed [4]. Another review article analyzed 12 in vitro, 8 in vivo, and 2 human studies focused on the use of probiotics to manage infected wounds. They found preliminary evidence supporting the effectiveness of specific probiotic strains. Lactobacillus plantarum was the most studied strain [155]. A similar literature review looked at 34 studies analyzing probiotics’ effects in human, animal, and in-vitro settings. The review article discussed the various benefits provided by probiotics. These included its inhibition of pathogens, ability to decrease the risk of infections, and properties to promote wound healing without any observable side effects [156]. Research into synbiotic therapies, which is a combination of probiotics and prebiotics, has also shown the potential to fight infections. A systematic review of synbiotics’ use in postoperative settings established that synbiotic therapy is the best regimen for reducing surgical site infections [157]. Most meta-analysis and review studies have found probiotics to be a safe and effective treatment with the potential to treat chronic wounds. Despite this, a significant gap exists in the literature, as large-scale and long-term clinical trials are absent. Further examination in the field of probiotics can provide more explanations of the mechanisms behind its effectiveness while also looking to improve its utility and practicality. Future research may also seek to bolster the evidence for probiotics’ safety as a chronic wound treatment. Figure 3 summarizes the conclusions from each of these review and meta-analyses.

The effects of probiotic use in vulnerable populations such as the critically ill, immunocompromised, or hospitalized patients is an imperative topic that must be considered if probiotics are going to become a widespread treatment [158,159]. Immunocompromised populations are at an increased risk of infection and are susceptible to septic conditions. As such, the introduction of a live microorganism that has the ability to acquire and transfer genes may pose a threat to immunocompromised individuals [159,160]. This indicates the need for more long-term studies with this specific population. Critically ill patients and those hospitalized are also at risk, as probiotic–host interactions are not completely understood. Moreover, some illnesses can affect the immune system and other biological processes, such as the amount of oxidative stress. Such processes are also influenced by probiotics, meaning that, when used in patients with these illnesses, interactions are unknown [159,161]. Overall, the lack of knowledge behind the actions of probiotics as well as the absence of long-term studies focused on vulnerable populations, are two limitations that may need to be resolved before broad clinical use.

## 4. Acetic Acid

Acetic acid is a weak organic acid that has been used as a topical treatment in the field of medicine for the last 6000 years [162,163]. Beginning in 1916, this antiseptic was documented to treat war-wound infections. Since then, it has been studied for its use as an effective present-day topical treatment [162]. Acidic wound environments can provide several benefits contributing to wound healing including the control of infections, protease activity inhibition, release of oxygen, toxicity of bacterial metabolites, and epithelialization and angiogenesis of tissue [164]. With the ever-growing rise of multidrug-resistant pathogens and the need for a simple, inexpensive solution to infected wounds, acetic acid treatments have recently seen renewed interest within the scientific community.

**Figure 4:**
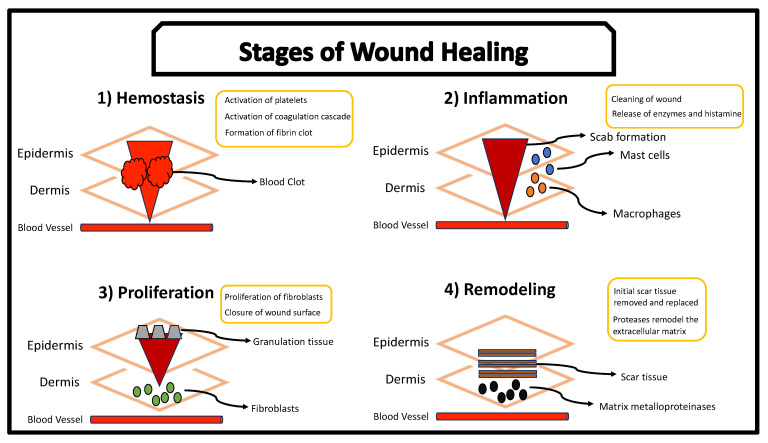
Illustration of the 4 stages of wound healing and some characteristics of each stage.

It has been documented that topical applications of higher concentrations of acetic acid can cause adverse side effects such as pain and itching [5,165,166]. Lower concentrations of acetic acid are less likely to cause side effects. Furthermore, a lower concentration of acetic acid may lead to a lower intensity of unpleasant olfactory stimulation that is usually emitted from the antiseptic. As such, acetic acid’s minimum inhibitory concentration of *PA* is a relevant topic of interest. Several studies have used acetic acid in concentrations less than 1% to inhibit *PA* or promote wound healing [162,163,165,167,168,169]. The majority of these studies demonstrate that *PA* can be completely eliminated using an acetic acid concentration as low as 0.146% to 0.30% [163,169]. However, most of these studies investigated a low acetic acid concentration and its effects on *PA* through in vitro experimentation. Chronic wounds are widely complex and evolving with many factors influencing the microenvironment and the microorganisms within [52,53,126]. Furthermore, wound healing is a highly convoluted process with an abundance of cellular mechanisms involved [53]. Figure 4 represents steps in the wound healing process. As such, further in vivo experimentations are needed to expand our understanding behind acetic acid’s mechanisms and interactions within a chronic infected wound.

Other studies have demonstrated the use of 1% acetic acid on infected human wounds [5,162,170,171,172]. One prospective study investigated the functionality of 1% acetic acid as the sole antimicrobial treatment for *PA*-infected wounds. The authors used saline as a control and followed 32 patients with chronic wounds over 6 months. The study found that 1% acetic acid eliminated *PA* infections from wounds 7 days earlier than the saline group. The study reported an average of a 5-day treatment period for the antiseptic to eliminate the bacterial infection from the chronic wound. With a low dose of acetic acid used, no major side effects were observed [5]. The study was not randomized or controlled, prompting the need for further investigation into the use of acetic acid in a clinical setting. Furthermore, a saline wound dressing is not an appropriate comparison, as a more up-to-date wound dressing would be a more effective control to evaluate acetic acid. The efficacy of acetic acid compared to the gold standard of wound treatments has not been fleshed out. Furthermore, the field of acetic acid research lacks randomized controlled studies that would help bolster acetic acid’s claim as a standalone, antibiotic-free treatment. However, through these various case studies and prospective studies, a 1% acetic acid concentration has been shown to be a safe and effective treatment. Most of these studies reported a treatment length between a few days and a couple of weeks [5,162,170,171,172]. This is around the same amount of time it takes for a course of antibiotics to eliminate *PA* [173]. Faster treatment times are considered to be more cost-effective, especially in the case of chronic wounds [174]. Results from a study found that low concentrations of acetic acid required a longer treatment time compared to higher concentrations when attempting to eliminate bacteria [169]. As such, higher concentration regimens should not be ignored, and may be worthwhile to consider in future studies.

A potential drawback of acetic acid is the frequent attention and dressing changes that are required. This can increase the economic burden associated with wound care [175,176]. However, acetic acid has been shown to be effective in treating multidrug-resistant strains of *PA* [169]. If it is effective at treating wounds that are not responsive to other treatments, acetic acid may help decrease the length of treatment and therefore the economic burden of the wound.

With attempts to narrow down an optimal acetic acid concentration for treatment use, a study set up experiments to determine the antibacterial activity of acetic acid at different concentrations. The study used *PA* along with other bacterial strains. In vivo experimentation showed that acetic acid concentrations from 5% to 0.31% effectively eradicated Gram-negative bacteria including *PA*. This study further noted that acetic acid at a 2.5% concentration had been used to reduce bacterial infections in vivo while still being tolerable for patients [163]. Similar studies concluded that 2.5–3% acetic acid treatments of infected burn wounds were well-tolerated and led to the complete elimination of multidrug-resistant *PA* and *PA*-producing biofilms [166,177]. One of these studies also suggested 3% as an ideal concentration that did not result in adverse side effects common in higher concentrations such as pain and itching [166]. While varying concentrations of acetic acid have been investigated, several factors should be considered when finding an ideal concentration including its efficacy, tolerability, length of treatment, cost-effectiveness, ability to kill multidrug-resistant strains, and compatibility with other treatments. Table 3 displays relevant acidic acid studies and results.

Interestingly, it has been found that a low pH is necessary for acetic acid to eliminate *PA* [162,163]. Specifically, acetic acid in its non-dissociated form, which occurs at a pH below 4.76, is deadly to *PA.* A low pH or an acidic environment alone does not eradicate *PA*. This was investigated by treating two cultures of the bacterium. One culture was treated with acetic acid at a pH below 4.76, while the other was treated with HCl at the same pH. HCl treatments at low pH levels did not eliminate any of the PA cultures, indicating that an acetic environment did not affect the pathogen. On the other hand, the acetic acid treatment at a low pH eliminated the *PA* culture [162,163].

The same study investigated the effect of acetic acid on cultured *PA* biofilms in a clinical patient setting. In the in-vitro experiments, 0.5% and 1% acetic acid treatments were found to be effective in completely eradicating *PA*. Sodium acetate, a dry source of acetic acid, was also discovered to be effective in eliminating the bacterium’s biofilm. For clinical experimentation, this study used acetic acid in combination with negative pressure wound therapy [162]. Research has shown that acetic acid may work well alongside negative pressure wound therapy [162,171]. While acetic acid works to eliminate bacterial infections, the use of negative pressure wound therapy gets rid of exudates, increases blood supply, and boosts collagen synthesis, all of which are beneficial to wound healing [178,179]. Many studies have demonstrated that negative pressure therapy resulted in faster healing times and is more cost-effective than other conventional therapies [178,180,181,182]. Negative pressure wound therapy combined with acetic acid may provide a simple, easily accessible, yet potent and cost-effective treatment to heal infected wounds [162,171].

On that note, acetic acid treatments for chronic wounds may work well when combined with regenerative therapies. While conventional therapies such as acetic acid work to control infections, regenerative wound healing techniques are employed to restore the skin and reestablish skin tissue. A few examples of regenerative therapies include negative pressure therapy, modern wound dressings such as hydrogel, and topical drug and growth factor deliveries [183,184,185]. A multiple case series reported using an acetic acid dressing until it eliminated signs of infection from the wound. This dressing was then replaced by a collagen or petrolatum dressing which functioned as a regenerative wound therapy to help achieve complete wound closure [167]. As stated earlier, acetic acid use alongside negative pressure wound therapy has been demonstrated by various studies [162,171]. If acetic acid was to be used in combination with a regenerative therapy technique, interactions may exist between compounds, suggesting the need for precautions and for more research to explore potential synergies. For example, collagen molecules are documented to be affected by acetic acid [186,187].

**Table 3:** Acetic acid (AA) studies involving the treatment of *PA*.

Acetic Acid Concentration	Type of Study	Length of Treatment	Results	Source
0.01–5%	In vitro	24 h	A minimum inhibitory concentration of 0.146% AA to kill MDR PAW1 strain of *PA*; 2× and 4× the minimum inhibitory concentration of AA caused rapid (within 5 min) elimination of *PA*	[169]
<0.10%, 0.16%, 0.31%	In vitro	3 h in 200 μL of AA	0.31% AA prevented planktonic and biofilm growth of 9 out of 9 different isolates of *PA*; <0.10% and 0.16% led to the prevention of biofilm formation in only some of the *PA* isolates	[163]
0.25%	Case study (*n* = 2)	Twice daily	Progressed wound healing and led to complete wound closure	[167]
0.50%	In vitro	24 h	0.50% eradicated *PA* biofilm	[168]
0.50%, 1%	In vitro	24 h	0.50% and 1% AA completely eradicated mature biofilm of *PA*; pH of acetic acid must be below 4.76 to be effective against *PA*	[162]
0.50–5%	Case study (*n* = 16)	7–14 days, 15 min twice daily	Elimination of *PA* from 14 of 16 patients after 2 weeks	[165]
1%	Prospective randomized controlled clinical trial (*n* = 32)	3–11 days, twice daily	1% AA treatment eliminated *PA* in chronic infections, trauma infections, and burn infections; AA eliminated *PA* faster than those treated with normal saline dressings	[5]
1%	Case study (*n* = 3)	5–12 days, 6 × 20 min per day	1% AA in combination with negative pressure wound therapy led to the promotion of wound healing	[162]
1%	Prospective Study (*n* = 72)	10–14 days	1% AA cleared *PA* infections from 65 of 72 patients	[170]
1%	Case Study (*n* = 3)	21 days, twice daily	1% AA with negative pressure wound therapy diminished wound size and showed less evidence of infection	[171]
1%	Prospective Study (*n* = 100)	7–21 days	1% AA eliminated all bacteria including *PA* (found in 40% of wounds) after 21 days; Decrease in wound size and inflammation; Overall signs of wound healing	[172]
3%	In vitro	5, 30, and 60 min in 9.9ml of AA	*PA* was eliminated after 5, 30, and 60 min of incubation	[166]
3%, 5%	Case study (*n* = 7)	2–12 days, daily application	3% AA eliminated *PA* in 6 out of 7 patients; 5% AA eliminated *PA* from 1 patient with a perinephric abscess	[188]

There is a lack of large-scale randomized controlled clinical trials investigating the use of acetic acid to treat *PA*-infected chronic wounds [177]. Several ex-vivo clinical observations and small-scale clinical studies suggest that acetic acid may be an effective antibiotic-free treatment that can have antimicrobial effects on *PA*-infected wounds [5,162,163,166,189]. A consensus on chronic wound antiseptics written in 2018 also listed acetic acid as a promising prospect in fighting *PA* infections [190]. The field of acetic acid treatments needs larger controlled in-vivo studies to further investigate an ideal treatment concentration as well as acetic acid’s overall practical use in clinical settings. Future research may also examine dry sources of acetic acid and acidic acid’s use as an adjuvant or concurrent treatment.

As acetic acid is readily available, inexpensive, and relatively non-toxic, it offers a simple but effective way to treat *PA*-infected chronic wounds. Silver-based wound dressings are common in addressing the antiseptic management of burn wounds but are not effective against *PA* [191,192]. As many studies have demonstrated the effectiveness of acetic acid against *PA* pathogens, acetic acid dressings may offer an alternative to a silver-based wound dressing. A study demonstrated that an antiseptic acetic acid matrix was a more efficient treatment for Gram-negative bacteria compared to silver-based therapies [192]. However, more robust investigations comparing the two wound dressings would aid in determining each’s advantages and disadvantages.

In developing countries, 1–2% of the population will have a chronic wound in their lifetime [193]. With chronic wounds remaining prevalent in countries with limited resources, a topical antiseptic such as acetic acid can potentially offer an accessible, easy-to-use, and cost-effective treatment for *PA* infections that does not require the use of antibiotics. Acetic acid may also be used in combination with other therapies to eliminate infection while also supporting the overall wound-healing process. Finally, acetic acid may be implemented in scenarios where bacterial infections with multidrug-resistant *PA* do not respond to other conventional treatments [5,162,169,170,171,172].

## 5. Essential Oils

Essential oils (EOs) are mixtures produced by aromatic plants and herbs. Of the roughly 3000 recognized EOs, 300 are generally recognized as safe by the Food and Drug Administration (FDA), and many have historically been used in food safety [194,195]. While EOs have long been used in folk medicine [196], there has been a revived interest in some of these natural products for their antibacterial and antiviral properties [197,198,199]. Essential oils are therefore included in this review of non-antibiotic methods of treating bacterial infections. More relevant to the context of this review is the possibility to use essential oils as a treatment for antibiotic-resistant *PA* [200]. If essential oils are found to be effective against *PA,* they could prove a potential alternative to antibiotics. To that end, one study tested four common essential oils— tea tree, thyme, sage, and eucalyptus—on *PA* strains isolated from hospital infections and wastewaters. These bacterial strands were further categorized based on their level of antibiotic resistance using the disc-diffusion method. This study found that 48% of the 36 strands were antibiotic-resistant. The strains of *PA* were then exposed to the four EOs and the researchers found average minimum inhibitory concentrations of 0.697%, 0.349%, 4.688%, and 2.726% for tea tree, thyme, sage, and eucalyptus, respectively [201]. Overall, the researchers found thymine to be the most effective EO against *PA*. Thyme’s chemical properties are responsible for its antibacterial activities. Due to the acidic nature of the thymol hydroxyl group, this compound forms hydrogen bonds with the active site of enzymes [198]. Several species of thyme have been demonstrated to display growth inhibition against *PA* including *Thymus vulgaris, T. algeriensis*, and *T. serpyllum* [201,202]. Moreover, thymus EOs may be effective in killing *PA* [203]. Besides thyme, cinnamaldehyde, a component of cinnamon and cinnamon bark EOs, has stood out as one of the most effective EOs displaying antibacterial activity [203,204,205,206]. Cinnamaldehyde is believed to fight *PA* by disrupting biofilm formation, which likely occurs through several mechanisms [204,207]. Furthermore, cinnamon EO may be an important component in various approaches to wound treatment. Interestingly, cinnamon EO has been incorporated into a nanofibrous scaffold which led to an improvement of the wound dressing’s antibacterial activity and biocompatibility [208]. Cinnamon leaf EO has also been combined with nitrocellulose, castor oil, ethanol, and ethyl acetate to form a film-forming gel. This gel can act on the skin and deliver anti-inflammatory and antibacterial effects to wounds [209]. At the time of writing, these are two very new wound treatment methods that promise innovation in this field. Due to cinnamon’s elevated effectiveness over other EOs, the use of cinnamon in novel wound dressings may lead to powerful synergies.

Another favorable EO is oregano oil, which has been shown to be effective against multidrug-resistant strains of *PA* [194,210]. A study found minimum inhibitory concentration of oregano oil between 0.16–0.56 mg/mL, depending upon the strain of *PA.* The researchers used mouse skin as a test and found no negative reaction to oregano oil at 10 mg/mL of concentration and three days of exposure. This concentration is far greater than necessary to eliminate the bacteria. As side effects of EOs are commonly connected to the dosage used [211], the lack of skin irritation even at high concentrations shows oregano oil to be a safe treatment [194]. Moreover, oregano oil has been demonstrated to exhibit anti-inflammatory properties [212,213]. A study also found that after 20 cycles of treatment, there was no significant difference in the effectiveness of the oregano oil as a treatment suggesting that oregano oil use does not lead to resistant strains. However, this doesn’t mean that bacterial resistance to oregano is impossible [194].

There is a risk that frequent and common use of EOs leads to resistance. For example, *PA* has specifically shown an increased tolerance to tea tree EO compounds [211]. The reality of this risk remains largely unknown and unaddressed, with no long-term studies existing. As such, further investigation into this topic would yield important information about EOs’ potential as a treatment. Further aspects of EO use should be taken into consideration, including any adverse side effects that it may produce. Contact allergies, dermatitis, stomatitis, and ototoxicity are some side effects associated with EOs [211,214]. Oregano EO has been demonstrated to be nontoxic and safe to use on human keratinocytes [215]. Furthermore, topical application of thyme has been demonstrated to be safe, without any side effects on patients [216]. Exploration into potential side effects associated with the specific use of EOs on chronic wounds would be appropriate.

EO’s mechanisms of action remain largely unknown, and, as such, the results of studies using EOs as treatments are difficult to interpret. However, investigation into the utility of EOs to fight bacteria suggests that EOs may play a role in degrading the cell wall and disrupting the cell membrane. This affects the membrane’s structure and function and is toxic to the bacteria due to cytoplasm coagulation, damage of membrane proteins, leakage of cell content, and interference of proton motive force and ATP synthesis [217].

## 6. Discussion

As discussed in this review, a wide range of treatments are currently being researched for chronic wounds with *PA* infections. Wounds that are infected with antibiotic-resistant and multidrug-resistant bacteria are difficult to treat, which has pushed the medical world to find solutions to the fight against these opportunistic pathogens. As infected chronic wounds remain a prevalent complication for many, and with the growth of antibiotic-resistant strains, the need for antibiotic-free treatments now and for the future is as large as ever. Photodynamic therapy is a unique radiation treatment that utilizes photosensitizers to create reactive oxygen species to eliminate pathogens. Various compounds can act as a photosensitizer, and each may offer different properties that may be beneficial in certain applications. By itself, or in combination with other therapies such as PTT or a pulsed electric field, research on PDT as an antimicrobial treatment is growing in popularity. Further research into this therapy might help determine an optimal photosensitizer and concentration to promote the healing of chronic wounds and the elimination of infections. Several compounds also show promise in improving the efficiency of PDT, which also may be a future topic of research. Moreover, while some mechanisms behind PDT’s wound-healing properties are known, further investigation may be warranted to expand our understanding of the therapy. Probiotic treatments are another distinct area of research that makes use of natural microorganisms to fight off infection and promote wound healing. Additionally, probiotics have the potential to be a component of modern state-of-the-art wound dressings. Probiotic treatment may also be utilized through exogenous, oral, or topical applications. Future studies may attempt to advance knowledge of the mechanisms behind probiotic treatments, as many interactions between beneficial bacteria and the human body remain unclear. A deeper understanding of probiotics could improve the effectiveness and safety of the treatments. Further advancements may utilize mixtures of probiotics and investigate some of their many strains, with the hope that they can eliminate multidrug-resistant strains of *PA*. Acetic acid is an inexpensive and widely accessible treatment with promising results that represents a simple antiseptic topical treatment for bacteria-infected chronic wounds. Various concentrations of acetic acid have been shown to be effective as a treatment, with little to no side effects occurring in patients. More data from randomized large-scale in vivo controlled clinical studies are needed to clarify acetic acid’s usefulness and practicality. Especially in countries with limited resources, acetic acid may offer an accessible therapy for *PA* and the promotion of wound healing. Essential oils such as cinnamon and thyme demonstrate inhibitory effects on *PA*. Future research may look to continue demonstrating essential oil use in clinical practice and comparing its effectiveness to other topical treatments such as acetic acid. 

It should be noted that PDT, probiotics, acetic acid, and essential oils only represent a small slice of the research into antibiotic-free treatments that are being used for chronic wounds. Furthermore, many of the studies discussed in this paper are case studies, and results demonstrated by such are not statistically significant. Outcomes from these studies should be approached with caution, as more research is needed to further validate conclusions drawn by these works. With that being said, our scientific knowledge is ever-growing and the medical research world is rapidly changing. Past and present ventures into these treatments, as discussed in this manuscript, indicate that more intensive and exhaustive efforts in these fields may provide significant results.

## 7. Conclusions

Photodynamic therapy, probiotics, acetic acid, and essential oils are a few of the promising antibiotic-free treatments on the rise in the medical research world that provide effective means of eliminating biofilm-forming *PA* in chronic wounds. In today’s world, with antibiotic-resistant bacteria causing increasing concern and complications in treatment, finding and exploring novel methods to heal chronic wounds is an important priority that should be properly investigated. This review article summarized relevant studies across pertinent fields of research with the hope of providing a concise review of the current state of potential treatments for chronic wounds. Further studies on this subject may continue striving to find an effective and practical ideal treatment that can be applied in widespread clinical scenarios.

## Figures and Tables

**Table 1:** ALA concentrations and their associated positive results, negative results, and limitations.

ALA Concentration	Length of Treatment	Positive Results (Observed in at Least Half the Subjects)	Negative Results (Observed in at Least Half the Subjects)	Limitations	Sources
0.5%	Once every 24 h for 1 month	Reduction in ulcer area, acceleration of wound healing	Fluctuations in blood tests	Small sample size, reduction in bacterial count in less than half of the subjects, lack of controls, inconsistent results	[41]
0.5%	Daily for 13 days	Reduction in biofilm production, acceleration of wound healing, acceleration of epithelialization	None	Animal mouse model	[42]
2%	One to three treatments over the course of 3 months	Elimination of bacteria after one treatment, healing of the ulcer, no recurrence of infection	None	Small sample size, only one subject had a control ulcer, long healing time, case study	[40]
20%	Once a week for 2 weeks [36] Once a week until wound healed [37] Once a week for up to 3 weeks [39]	Reduction in bacteria levels [36,37], reduction in ulcer area [36,37,39], recurrence of infection [37], induced proinflammatory mediators and recruitment of immune cells [39]	Differing degrees of pain, redness, and swelling around the ulcer area [36,37,39]	No long-term follow-ups [36], small sample size [37], lack of controls [37,39]	[36,37,39]
1.408 mol/L	14 days	Reduction of *PA* load, promotion of wound healing, regulation of inflammatory factors, collagen remodeling, and macrophages	None	Animal mouse model	[44]

## Data Availability

Not applicable.
